# Psychosocial effects of gender-based violence among women in Vhembe district: A qualitative study

**DOI:** 10.4102/sajpsychiatry.v29i0.2012

**Published:** 2023-08-22

**Authors:** Rodney Rikhotso, Thinavhuyo R. Netangaheni, Nongiwe L. Mhlanga

**Affiliations:** 1 Department of Social Work, Faculty of Human Sciences, University of South Africa, Pretoria, South Africa; 2 Department of Health Studies, Faculty of Human Sciences, University of South Africa, Pretoria, South Africa

**Keywords:** gender-based violence, psychosocial effects, Vhembe district, women, effects, psychosocial

## Abstract

**Background:**

The phenomenon of gender-based violence is a pertinent social problem in South Africa. The fear of reporting gender-based violence contributes to its continuation, marginalisation and silencing of victims.

**Objectives:**

The study sought to explore the psychosocial effects of gender-based violence among women in Vhembe district.

**Methods:**

An exploratory phenomenological research design was used and sampling was performed purposively from a population of women who experienced gender-based violence in a low-resource, rural setting of Vhembe district. Semi-structured telephonic interviews were used as the main method of data collection after permissions and informed consent were sought for conducting the study. Thematic content analysis was applied to convert the participants’ statements into a meaningful framework to derive the findings.

**Results:**

A total of 15 participants aged from 19 to 35 years participated in the study. Their psychosocial experiences of gender-based violence were depression, worthlessness, social isolation and anger directed towards children.

**Conclusion:**

This research confirms that gender-based violence remains one of the most challenging problems associated with mental health problems in Vhembe district. It affirms the need to focus on awareness in rural areas afflicted by patriarchal attitudes, norms and stereotypes. Gender-based violence should to be viewed as human rights violation for victims’ protection.

**Contribution:**

The study contributes to the body of knowledge on the experiences of gender-based violence among marginalised women from rural areas.

## Introduction

Gender-based violence (GBV) is violence directed towards other people and is subject to various conceptual interpretations and contextual applications.^1^ Gender-based violence is founded on gender inequality with most victims being women.^2^ It is a serious public health and human rights issue, and its manifestations are classified according to emotional, physical, social, psychological, sexual, economic and domestic forms.^3^ It encompasses intimate partner violence (IPV), which is sexual or physical violence committed by a current or previous partner after the age of 15 years.^3^

The World Health Organization (WHO) states that males are more likely to perpetrate GBV while women and girls of all ages are victims.^3^ Globally, by the end of 2021, IPV resulted in at least 137 femicides daily.^4^ In 2019, at least ‘243 million women and girls aged 15–49 across the world’ were victims of GBV and suffered from the mental, physical, spiritual, sexual and/or reproductive health aftermaths.^5^ In the South African context, there was a total number of 224 912 general crimes against children and women (179 683 against women and 45 229 against children), during the 2018 and 2019 periods alone. These statistics were the highest in the world, characterising South Africa as ‘the rape capital of the world’.^6^ In Limpopo province, studies by the Thohoyandou Victim Empowerment Programme (TVEP) have established that the Vhembe District Municipality (VDM) has the highest reported cases of domestic violence in Limpopo province.^7,8^ Statistics on GBV in South Africa illustrate its high prevalence with the general public calling incessantly for interventions to prevent and mitigate GBV. As such, this study is based on the problem of GBV and is founded on the effects of GBV in the VDM.

Several factors have been alluded to the perpetration of GBV. In a study conducted in 12 African countries, the authors concluded that absence of laws on GBV, alcohol consumption, male dominance, women’s attitudes after the perpetration of GBV and their empowerment predict GBV.^9^ In a systematic review, Van Daalen et al. concluded that GBV was related to food insecurity, economic hardship and disruption of infrastructure because of extreme weather conditions.^10^ The review by Van Daalen et al. further observed that GBV is related to harmful cultural or traditional practices against women such as early marriages.^10^ The authors notice the cyclical effects of GBV by highlighting that GBV results in women transferring the violence and anger towards children.^10^

Several studies explored the mental health issues and behavioural disturbances among victims of GBV. A survey with 273 respondents, conducted in Australia, concluded that GBV results in a complexity of mental health challenges that include social isolation which worsens the effects of GBV, as victims are unable to seek help and reduce occurrence of GBV.^11^ In a narrative review of literature, in the United States, GBV was also associated with increased childhood exposure to trauma.^12^ In Africa, a survey with 209 women in Kenya concluded that GBV resulted in anxiety, depression and post-traumatic stress disorder in women and girls.^13^ In another survey with 283 respondents conducted in Kenya, the authors found that GBV was associated with poor mental health, high-risk sexual behaviour and sexually transmitted infections.^14^ In addition, GBV resulted in disordered alcohol usage among women.^14^ A Nigerian study revealed that 31% of the participants agreed that women suffered GBV because they were viewed as ‘inferior to males, incompetent and worthless’.^15^ This Nigerian study further concluded that women were not allowed to associate with male relatives or male friends.^15^ On this issue of social isolation, South African statistics reveal that most women (60%) did not report GBV while 40% reported to law enforcement.^16^ Also, in South Africa, a longitudinal study with a sample size of 415 participants found that GBV results in depression and suicidal ideation.^17^ These issues highlight the seriousness of GBV, should prompt society to respond to these victims and increase awareness on the need to prevent GBV. Therefore, the purpose of the study is to explore the psychosocial effects of GBV among women in Vhembe district.

## Research methods and design

This study used a qualitative approach to explore the psychosocial effects of GBV among marginalised women in Vhembe district, Limpopo province.

### Study design

This study opted for the phenomenological research design to understand the phenomenon of GBV as constructed by the participants themselves in their own familiar ecological surroundings.^18^ Phenomenological designs enable the search and establishment of knowledge, truth or reality of a phenomenon as socially constructed products of the affected individuals’ experiences and perspectives concerning that very phenomenon.^19^ The study utilised an interpretivist paradigm that acknowledges that the culture and context are different among research participants and seeks to interpret such subjective experiences among women experiencing GBV in Vhembe district.^18^

### Setting

The researchers conveniently selected to undertake the study in the N’wamatatani and Hlanganani rural informal settlements located in the VDM, one of the five districts in Limpopo province. The VDM has a population of about 1.385 million residents.^20^ The district is largely populated by the Venda, Tsonga, Bapedi and Afrikaners.^20^ The dominant Vhembe district languages are Tshivenda and Xitsonga, followed by Sepedi, Afrikaans and other minority languages of migrants Zimbabwe and Mozambique.^20^

### Study population and sampling strategy

The study population consisted of women victims of GBV in the study setting. Purposive sampling was conducted whereby participants known to the Department of Social Development Area Social Worker who worked with female GBV victims were invited to participate. The inclusion criteria included all females aged 19–35 years who directly experienced GBV; women experiencing GBV who were residents of N’wamatatani and/or Hlanganani informal settlement in the VDM; and women who were willing to participate voluntarily and be audio-recorded through telephonic interviews. The study included all women till data saturation, whereby no additional new information could be solicited from participants upon further interviewing more participants (data saturation was reached at 15 participants).

### Data collection

The principal investigator (RR) conducted the 15 semi-structured, in-depth interviews telephonically, each lasting 30 min–45 min. Open-ended questions were used during data collection, which enabled the researcher to implement probing questions and to elicit participants’ spontaneous or unhindered responses on GBV. The principal researcher (RR) asked the participants questions and probed for clarity in cases of misunderstanding and for more insight, despite the telephonic interviews that were performed in adherence to affiliated institutions’ coronavirus disease 2019 (COVID-19) guidelines at the time of the study. For all these telephonic interviews, the participants were requested to ensure that they were at a quiet place that would not cause distractions to them. They were informed that arrangements had been made with the Area Social Worker in case they needed further interventions or assistance during the interviews because of questions that could disturb them emotionally and/or psychologically. All 15 participants were interviewed within a space of 5 days between 15 March 2021 and 19 March 2021. Three participants were interviewed on each day. The researcher sought clarity from the participants concerning responses that the researcher did not understand. The interviews were conducted in Xitsonga and English, as it was easy for the participants to freely express themselves.

### Data analysis

Thematic analysis was applied in this study, founded on the procedure proposed by Braun and Clark in 2006.^19^ This involved all English interviews being transcribed, with the Xitsonga interviews firstly being translated and then transcribed to English. To ensure rigour of the translated data, the researcher checked the correctness of the transcriptions by asking a participant who could speak both Xitsonga and English to verify the correctness of the transcripts. The transcripts were examined for themes, using Braun and Clark’s six steps for thematic analysis.^19^ These steps included generating codes from the initial data collected, searching for themes in the transcribed data, reviewing the themes which had been found, and finally defining and naming the themes.^19^ The themes generated from the analysis were corroborated by the participants to ensure trustworthiness. Following this corroboration, the themes were presented in a narrative format.

The researcher ensured the study’s trustworthiness by accurately recording all procedures taken during the study to enable auditing and verifying the results of the study with the study participants.^19^ The researcher reflected on their role as a social worker who works with women victims of GBV and maintained objectivity as participants shared their experiences through use of an interview guide to ensure that questions asked were related to the research, thus minimising the researcher imposing their beliefs.

### Ethical considerations

Ethical clearance for this study was approved by the College of Human Sciences Research Ethics Review Committee at the University of South Africa. Prior to the commencement of data collection, all the participants signed the consent forms with the assistance of the Area Social Worker. Participants were notified about their right to participate or decline before any involvement in the study. The researcher further explained that their involvement would be in the form of answering the researcher’s questions orally. Prior to the commencement of the interviews, the researcher fully disclosed what the study entails and the rights of the participants (2020-PsyREC-56712618).

## Results

### Participants’ biographical profiles

Fifteen female participants formed part of the study, all of whom were black, with 14 South African nationals and 1 Mozambiquan expatriate. They were all aged from 19 to 35 years and Xitsonga speaking and had experienced GBV in the form of IPV. Eight of the participants were married, five were single, one was divorced and one was widowed. Eight of the participants still lived with their partners while seven no longer lived with their partners. All participants resided in informal housing of Hlanganani and N’wamatatani settlements in the VDM where the study was undertaken.

### Key findings

From the phenomenological analysis, four main themes emerged that described the psychosocial effects of GBV on women in the Vhembe district. The first main theme was an effect of ‘worthlessness’ as a result of GBV. The second effect of GBV among women in Vhembe district was ‘social isolation’. The third was ‘depression’ as a consequence of GBV. The last theme was that GBV had a psychosocial effect of causing ‘anger towards children’ among women in the Vhembe district.

The first theme was the issue of ‘experience of worthlessness’ associated with GBV. The issue of experiencing worthlessness among victims of GBV was expressed by five participants. This response was elicited from the discussion on the topic, GBV, and its emotional effects. The participants described their experiences of feeling worthless without their husbands. From these shared experiences, participants highlighted they felt worthlessness because a bride price had been paid for them or they came from a poor background. Excerpts from participants C and I are as follows:

‘First it was emotional and then it escalated to physical violence. I was told that I am nothing without him and there are a lot of things I cannot achieve without him because I am from a poor family. Today I know how to wear a night dress because of him and that I came with nothing to the marriage … I most of the time feel worthless as a woman.’ (Participant C, 26 year old, female, single)

‘I think it is the reason that I stayed in the relationship for too long to a point where my husband realized that I will not leave him. Again, I think he ended up viewing me as his property because he paid lobola or dowry for me … The violence I suffered killed my self-esteem because of being told that I am useless.’ (Participant I, 27 year old, female, married)

The second theme to emerge was ‘experiencing social isolation’ as a consequence of GBV. This theme was elicited in response to the topic on family/friends being aware of the GBV. Participants A, G, K, L and M expressed that the social isolation was related to insecurities of their partners who suspected infidelity of the women. Participant N similarly revealed she feared her spouse who was insecure and obsessive to the extent of following to her place of work for the purpose of taking her home after work. The following excerpts from participants A and G illustrate the experiences of social isolation:

‘My husband was very insecure. He did not want me to associate with other people because of his jealous … That man isolated me from people that I cared about. He made me see no value in interacting with other people with the fear of being judged even though they did not know. I actually, lost interest in socialising with other people.’ (Participant A, 35 year old, female, divorced)

‘He is insecure due to his past relationships and very jealous. He does not allow me to interact with my friends freely over the phone. He assaults me when I am using my phone thinking that I am talking to other men. Alcohol abuse and trust issues are also the contributing factors.’ (Participant G, 23 year old, female, single)

The third theme to emerge was ‘depression was associated with GBV’. This theme was also elicited when discussing the topic on emotional consequences of GBV. The participants described how the effects of GBV became the source of their depression. Participant B noticed they became depressed after the GBV and when their partner took away their child. Participant K who was subjected to GBV similarly observed they had become depressed after they found out their daughter had been sexually abused. The theme is evidenced from the following excerpts from participants:

‘It destroyed me. I lost myself. There was a time where I felt that I was done with him. However, he spent more time with my family members drinking alcohol even after I left him. My family looked at me as the source of the problems we encountered and view him as an angel. I ended up being depressed after he took custody of my last-born baby. At work it affected me a lot because I would even see case dockets of women who were killed by their partners. I would cry before I go to work.’ (Participant B, 27 year old, female, married)

‘That abuse affected me so much because I always went back to work on with bruises and pain but what shattered me the most was that after the death of my husband my daughter told me that he used to rape her in my absence. I don’t know whether it was because she was his step-daughter or what. I was depressed for more than three years because of what he did given the fact that after his death I found out that I was HIV positive.’ (Participant K, 34 year old, female, widowed)

The fourth theme was that GBV is experienced through ‘anger towards children’. This is evidenced by responses from participants A, D and E. The issue of anger and irritability towards children emanated on discussing the topic, on whether participants had children and if the GBV occurs in the presence of the children; if the response was yes, a follow-up question on how the GBV affected the children was asked. Participants described how the GBV resulted in them becoming angry towards their children and would easily shout at them. Participants A and D elaborated that they would shout at the children for no apparent reasons. The excerpts from their responses are as follows:

‘The abuse I endured has changed me a lot. I have trust issues and I always snap at people every now and then. What pains me the most is that I even shout at my children for unnecessary things. Even in my current relationship I had to go for counselling with my boyfriend because I am always ready to defend myself.’ (Participant A, 35 year old, female, divorced)

‘This thing is really affecting me because you can find that sometimes I fight with children, taking my frustrations, my anger to the children. Like I told you that I fight back and I started to be somebody I don’t know. I also don’t understand myself. My children are suffering for what they don’t even know [*crying*].’ (Participant D 30 year old, female, married)

‘I am always unhappy and the way I treat my children shows that I am releasing that anger. I display a lot of anger towards other people. The abuse I suffered has changed me completely.’ (Participant E, 21 year old, female, married)

Notably, the GBV and anger resulted in children displaying disturbing behaviours towards other children as observed by Participant G who related that she had been asked to go to her child’s school following reports of bullying other children. The quotation to support this is as follows:

‘I am not allowed to go out with my friends and that affects me because I end up sitting at home with my son or with him when he is around and my sons sees all the violence and bullies other children at school, I was called to the school once because of the bullying.’ (Participant G, 23 year old, female, single)

## Discussion

Four themes were discussed, two of which formed part of the modus operandi of the abusive partner (worthlessness and social isolation) and two appeared to be effects of the violence (depression, irritability, anger towards their children). One participant also expressed concern over anger and abusive behaviour evident in her child, behaviour that she thought was a consequence of witnessing violence against her.

These findings are of particular interest as they present the effects of GBV in Vhembe district, which is a population underrepresented in literature on GBV, as well as confirm findings from other studies conducted in other parts of the world.

Worthlessness described by the participants may have been part of the abuse as the women were reminded by their abusers that they were nothing, and in some instances, the worthlessness was a sequela to the GBV. These feelings of worthlessness are also supported by a review conducted in 12 African countries, which found that women’s attitudes on GBV perpetuate GBV through feelings of worthlessness.^9^ Such feelings of worthlessness associated with GBV could also be a symptom of post-traumatic stress disorder described by a narrative literature review in the United States.^15^

The participants experienced social isolation that was imposed on them as part of the controlling or abusive behaviour of their partners or as a symptom of depression as they felt they were cut off from social contact. This social isolation is described by Fernández-Fillol et al.^21^ who observed that women who experience GBV exhibit signs of post-traumatic stress disorder, which manifests through social isolation. These findings are confirmed by a study conducted in Australia where the authors found that GBV results in social isolation.^11^ Similar findings were noticed from a study conducted in Nigeria, where GBV experiences included prohibition from socialisation with family and friends, as women were viewed as worthless.^15^ The authors from the Australian study further found that the social isolation made it difficult to provide help to GBV victims.^11^ This implication is drawn from the different characterisations of GBV such as financial, emotional, psychological and sexual, which may not be easily visible.^1^ Furthermore, the social isolation and consequential silence on GBV victims were evident in South Africa, where only 40% of cases are reported, warrants further studies on interventions that empower women and children to speak up and access psychosocial assistance.^16^

The participants shared their experiences of anger that they would direct towards the children. The issue of anger was also confirmed in a systematic mixed-methods review that GBV has negative ramifications for children.^10^ The anger towards children concluded in this study necessitates a holistic approach in low- to middle-income countries in the management of GBV, which provides for children of women who are victims of GBV to be cared for. The negative consequences of GBV affecting children could be as a result of difficulty in parenting or poor emotion regulation, which could be asked about in the International Trauma Questionnaire. With regard to social isolation, the finding implies that service providers may experience difficulty in recognising and providing women in need of healthcare or psychosocial support because of the GBV.

### Limitations

Despite the research objective being achieved, the study was limited by the methodological approach. The researchers used a qualitative approach with a small sample size, as such results cannot be generalised to all victims of GBV.

## Conclusion

The study sought to explore psychosocial effects of GBV among women in the VDM in Limpopo province. The study recognises that GBV is a global psychosocial problem that infringes on the rights of women and relegates women to inferior status in society. The determinants of GBV are varied, and as a consequence, the experiences of women vary across different cultures and communities. The study concluded that in VDM, women experienced psychosocial effects of depression, anger towards children, social isolation and worthlessness because of GBV. From these findings, a holistic approach to prevent and manage GBV is recommended. This approach should empower women to seek assistance mitigating the experiences of social isolation that results in depression and anger towards children, who also need assistance to manage effects of GBV. The study further corroborated several studies that empowerment through women’s employment together with a change in norms, attitudes and roles are critical interventions among marginalised women. Further studies in treatment approaches for GBV in rural communities are recommended.
